# Systemic and renal oxidative stress in the pathogenesis of hypertension: modulation of long-term control of arterial blood pressure by resveratrol

**DOI:** 10.3389/fphys.2014.00292

**Published:** 2014-08-05

**Authors:** Shereen M. Hamza, Jason R. B. Dyck

**Affiliations:** ^1^Department of Pediatrics, Cardiovascular Research Centre, University of AlbertaEdmonton, AB, Canada; ^2^Department of Pharmacology, Cardiovascular Research Centre, University of AlbertaEdmonton, AB, Canada

**Keywords:** resveratrol, pressure natriuresis, oxidative stress, renal dysfunction, hypertension

## Abstract

Hypertension affects over 25% of the global population and is associated with grave and often fatal complications that affect many organ systems. Although great advancements have been made in the clinical assessment and treatment of hypertension, the cause of hypertension in over 90% of these patients is unknown, which hampers the development of targeted and more effective treatment. The etiology of hypertension involves multiple pathological processes and organ systems, however one unifying feature of all of these contributing factors is oxidative stress. Once the body's natural anti-oxidant defense mechanisms are overwhelmed, reactive oxygen species (ROS) begin to accumulate in the tissues. ROS play important roles in normal regulation of many physiological processes, however in excess they are detrimental and cause widespread cell and tissue damage as well as derangements in many physiological processes. Thus, control of oxidative stress has become an attractive target for pharmacotherapy to prevent and manage hypertension. Resveratrol (trans-3,5,4′-Trihydroxystilbene) is a naturally occurring polyphenol which has anti-oxidant effects *in vivo*. Many studies have shown anti-hypertensive effects of resveratrol in different pre-clinical models of hypertension, via a multitude of mechanisms that include its function as an anti-oxidant. However, results have been mixed and in some cases resveratrol has no effect on blood pressure. This may be due to the heavy emphasis on peripheral vasodilator effects of resveratrol and virtually no investigation of its potential renal effects. This is particularly troubling in the arena of hypertension, where it is well known and accepted that the kidney plays an essential role in the long term regulation of arterial pressure and a vital role in the initiation, development and maintenance of chronic hypertension. It is thus the focus of this review to discuss the potential of resveratrol as an anti-hypertensive treatment via amelioration of oxidative stress within the framework of the fundamental physiological principles of long term regulation of arterial blood pressure.

## Introduction

Hypertension is a serious chronic condition that is a leading cause of morbidity and mortality worldwide (Lawes et al., 2008; Wilkins et al., 2010; Chobanian, 2011). It is associated with many potentially fatal complications that affect nearly every organ system in the body and remains a clinical problem (Kannel, 2000). Of importance, >90% of patients develop hypertension due to an unknown cause and thus current therapy is designed to control the major symptom of hypertension, high arterial blood pressure, as opposed to the root cause of the disease. Of these patients, another 20–30% are resistant to blood pressure reduction by currently available anti-hypertensive therapies (Calhoun et al., 2008). Although hypertension has been the subject of intense research focus for decades, its precise etiology remains unclear. This is due to the intricate and multifactorial nature of the disease (Montezano and Touyz, 2012), which is the product of interactions between genetic, physiological and environmental elements. Despite this complexity, there are several universally accepted facts regarding the pathogenesis of hypertension. In addition to accepted, but largely undefined genetic and environmental influences, other factors that are thought to contribute to chronically elevated arterial blood pressure include: (1) cardiac and vascular remodeling, (2) increased cardiac output and total peripheral resistance, (3) diminished production/responsivity to vasodilators, (4) inflammation, (5) immune reaction (Muller et al., 2011), (6) abnormal cell signaling (i.e., vascular, renal) (Belmonte and Blaxall, 2011), (7) elevated sympathetic nervous tone (Zubcevic et al., 2011), (8) arterial baroreceptor adaptation, (9) over activity of renin-angiotensin-aldosterone system (RAAS) (Savoia et al., 2011) and (10) renal dysfunction (Singh et al., 2010). Notwithstanding this vast diversity of hypertensive processes, a common theme amongst them is the increased presence (bioavailability) of reactive oxygen species known as oxidative stress (Montezano and Touyz, 2012).

Oxidative stress occurs when there is a relative imbalance between oxidant and anti-oxidant molecules in the body, leading to an accumulation of reactive oxygen species (ROS) (Puddu et al., 2008; Sugamura and Keaney, 2011). ROS are chemically active molecules that contain oxygen and are naturally formed as by-products of oxygen metabolism that occurs in normal physiology (Forman et al., 2008). Examples of ROS include: unstable free radicals (superoxide O^−^_2_; hydroxyl radical OH^−^) and non-free radicals (hydrogen peroxide H_2_O_2_; peroxynitrite ONOO-; hypochlorous acid HOCl) to name just a few. While often discussed in a negative context, ROS are important in normal cell signaling and maintenance of homeostasis (Jacob et al., 2011). Indeed, ROS are known to participate in gene activation and transcription and modulate signal transduction. However, in the cases where levels of ROS rise dramatically and exceed the body's endogenous anti-oxidant defense mechanisms, ROS can result in significant damage to cells and tissues (Montezano and Touyz, 2012). In addition, ROS reduce the bioavailability of the protective vasodilator nitric oxide (NO) and can modulate endothelial function, vascular tone and cardiac function (Rybalkin et al., 2002). In addition, ROS have been implicated in pathological processes such as inflammation, hypertrophy, apoptosis, fibrosis, and vessel rarefaction (Touyz, 2005; Forstermann, 2008). These processes are, in turn, involved in the development of endothelial dysfunction and cardiovascular remodeling that are characteristic of hypertension (Touyz, 2005; Forstermann, 2008). As such, a promising field of research has centered on the amelioration of oxidative stress, largely by application of anti-oxidant molecules in the prevention and treatment of hypertension.

Resveratrol (3,5,4′-trihydroxy-trans-stilbene) is a polyphenol molecule naturally produced in more than 70 plant species in response to environmental stress and in this setting, functions as a phytoalexin—preventing the proliferation of pathogens (Soleas et al., 1997). Resveratrol is found in high concentrations in rhubarb roots, blueberries, peanuts and particularly in the skin of red grapes (Baur and Sinclair, 2006) and their derivatives such as grape juice and red wine (Soleas et al., 1997). Considerable interest in resveratrol was initiated in 1992 with the demonstration of the cardioprotective effects of red wine (Siemann and Creasy, 1992). It was thought that resveratrol may be the key factor in the “French Paradox” in which the French population was observed to have a low incidence of cardiovascular disease despite consumption of a diet high in saturated fat (Renaud and de Lorgeril, 1992; Liu et al., 2007; Timmers et al., 2012). Resveratrol is a molecule with many targets and, as such, it produces many biological effects ranging from life-span extension and improved motor function to treatment of obesity (Baur et al., 2006; Harikumar and Aggarwal, 2008; Pirola and Frojdo, 2008).

Although the cardioprotective benefits of resveratrol are perhaps the best known and investigated (Catalgol et al., 2012), the function of resveratrol as an anti-oxidant agent have also made it an attractive target for the prevention and treatment of hypertension. Many studies utilizing resveratrol have since been completed in various animal models of hypertension and in a limited number of human clinical trials. However, the ability of resveratrol to produce sustained, long-term reductions in arterial pressure remains controversial, with even less impressive results in hypertensive humans (Pollack and Crandall, 2013). These mixed results may be due to several factors, one of which may be the heavy experimental emphasis on the peripheral vascular actions of resveratrol in the setting of hypertension. Because essential hypertension is associated with increased total peripheral resistance, many scientific investigations are focused on the factors that cause, and can thus ameliorate peripheral vasoconstriction. However, following the seminal work of Guyton and Coleman in the 1960's (Guyton et al., 1969), it is fundamentally known that the kidney is integral in the long term control of arterial pressure and that abnormalities in renal function play a key role in the pathogenesis of hypertension (Guyton, 1980). Unfortunately, there remains a paucity of information regarding the impact of resveratrol on renal function, particularly in the setting of hypertension. Since it is ultimately the interaction of peripheral and renal mechanisms that eventually produce chronic hypertension, it is the purpose of this review to discuss peripheral and putative renal actions of resveratrol in the setting of oxidative stress as a contributing mechanism to the pathogenesis of hypertension. In this discussion, physiological long-term regulation of arterial pressure will be reviewed and the necessity for understanding the modulation of renal function by resveratrol in order to effectively target treatment of hypertension will be emphasized.

## Nutrapharmacology of resveratrol

Considering the increasingly prominent role of oxidative stress in the pathogenesis of hypertension, enhancing anti-oxidant status is a reasonable treatment strategy and is indeed associated with a reduction of blood pressure. Oxidative stress is a multi-system phenomenon involving the heart, kidneys, nervous system, vasculature and immune system (Montezano and Touyz, 2012) and as such, anti-oxidant therapy with resveratrol can potentially target any or all of these systems. Resveratrol is able to reduce cardiovascular risk by inhibiting oxidative stress by blockade of ROS generation as well as regulation of vasodilator and vasoconstrictor production. Following oral ingestion of resveratrol, reabsorption is quite rapid in animals and humans, however, plasma levels are generally low due to the rapid conversion of resveratrol to metabolites which can then be rapidly excreted (Kroon et al., 2010). Generally the plasma half-life of resveratrol in humans is ~4–8 h at higher doses (~200–500 mg/kg) (Boocock et al., 2007). Because of this rapid metabolism, it is most likely that some degree of tissue accumulation of resveratrol (or one of its metabolites) has to sufficiently occur in order to produce therapeutic responses (Kroon et al., 2010). Limited evidence demonstrates that the highest resveratrol concentrations are found in those organs which are primarily involved in absorption, metabolism and elimination—namely duodenum, colon, liver and kidneys (Vitrac et al., 2003). The total concentration of resveratrol in the kidneys 3 h following ingestion of a single bolus dose reached 30 μM, demonstrating that resveratrol can indeed accumulate at supra-circulatory levels in key organs and thus has greater potential to cause meaningful biological effects (Vitrac et al., 2003). The finding that resveratrol accumulates in the kidney is particularly significant and further supports a direct renal effect of this treatment in the reported blood pressure lowering that is typically attributed to extra-renal actions of resveratrol. We will now focus on the essential role of the kidney in the development of hypertension and the modulation of long term arterial pressure by oxidative stress before discussing the mechanisms by which resveratrol may exert antihypertensive effects.

## Renal mechanisms of hypertension

The fundamental role of renal dysfunction in hypertension has been recognized as early as the mid-1800's at which time Bright noted small, shrunken and scarred kidneys associated with cardiac hypertrophy (Cowley, 2008). Goldblatt directly linked the kidney with chronic hypertension as did early experiments by Dahl (Dahl et al., 1974; Dahl and Heine, 1975; Cowley, 2008). In these studies, a kidney from a salt-sensitive and hypertensive rat was transplanted into a salt-resistant, normotensive rat, which subsequently produced hypertension in the resistant animal. Conversely, transplantation of a kidney from a normotensive, salt-resistant rat into a salt-sensitive rat prevented the expected rise in blood pressure (Dahl et al., 1974; Dahl and Heine, 1975). This phenomenon was later reproduced in genetic models of hypertension (Okamoto SHR, stroke-prone SHR, Milan hypertensive) (Bianchi et al., 1973, 1978, 1988; Kawabe et al., 1978; Rettig et al., 1989). These experiments demonstrated that hypertension “followed” the kidney and suggested that the kidney is the key link between genetic and environmental factors with respect to the regulation of blood pressure. The findings also support the notion that, at least in these models, blood pressure is dictated by the kidneys rather than the many systemic adaptations that are associated with the hypertension. Consistent with the pre-clinical studies, in humans, transplantation of a “normotensive” kidney into a hypertensive patient with renal failure has been shown to normalize blood pressure for the entire 4–5 year follow up period (Curtis et al., 1983). Thus, if factors external to the kidney caused the hypertension, blood pressure would rise over time in transplant recipients. However this was not the case and these findings support the view that essential hypertension is due to some abnormality in renal function.

In addition to these early kidney transplant studies, other animal models of hypertension also support a causal role of the kidney in hypertension (Hall, 1994). Over time, with the progression of hypertension, the original perturbation in renal function may be masked by compensatory mechanisms which attempt to normalize renal function. These changes involve cardiac and vascular adaptations which may become more apparent than the originating renal dysfunction and these changes are often the focus of hypertension research as a result. In fact, in the early stages of human hypertension, there is no clinically detectable change in renal function or neurohormonal perturbation (Hall, 1994), emphasizing the unknown cause of hypertension and perhaps accounting for the investigative gravitation toward extra-renal mechanisms of hypertension.

Key features in the pathogenesis of hypertension include an increase in cardiac output and total peripheral resistance. The kidney itself is a source of vasoactive mediators that can influence total peripheral resistance and cardiac function. Via renal afferent nerve activity, the kidney can also modulate sympathetic nervous outflow, which is an important controller of vascular tone (Kopp, 2011). In addition, the kidney can control blood pressure through regulation of blood volume. While vasoactive mediators and sympathetic tone play a critical role in the short-term regulation of arterial pressure, it is renal excretion of sodium and water which are essential for long-term control of arterial pressure and, ultimately, the development of chronic hypertension (Guyton, 1980, 1990a). Thus it is critical to briefly review the fundamental principles of the physiological regulation of blood volume and blood pressure.

### Pressure natriuresis and diuresis

Arterial pressure is simply expressed as cardiac output × total peripheral resistance. At first glance, one may focus on physiological factors that will directly modify cardiac and vascular function as the mechanism to treat hypertension. However, it is critical not to lose sight of the importance of renal excretion in the overall control of blood pressure. Long-term control of blood pressure involves complex mechanisms that differ from short-term control and involves control of body fluid volume (Guyton et al., 1969, 1976; Guyton, 1980; Cowley, 1992).

Pressure natriuresis and diuresis is an important mechanism for maintaining this “renal-body fluid feedback.” In the setting of normal kidney function, any increase in blood pressure will be matched with an equivalent increase in sodium and water excretion via pressure natriuresis and diuresis. As a result, extracellular fluid volume will progressively decrease, reducing venous return and cardiac output until blood pressure returns toward normal and the balance between fluid intake and output is re-established. Conversely, if blood pressure decreases, the kidneys will retain sodium and water which will expand the extracellular fluid volume until blood pressure is normalized. An important feature of the pressure natriuresis mechanism is modification by neurohormonal systems to augment or blunt the effect of blood pressure on sodium and water excretion [i.e., renal sympathetic nerve activity (RSNA); angiotensin II (Ang II), atrial natriuretic peptide (ANP)] (Guyton, 1980; Hall et al., 1986a,b). Another critical feature of pressure natriuresis is that this mechanism will continue to function until blood pressure is returned to the original set-point—the set-point determined by renal excretory capability (Guyton, 1980, 1990b). If normally functioning and intact, the kidney thus has unlimited potential to regulate blood volume and pressure, referred to as an “infinite gain” feedback control system (Guyton, 1990b). In other words, any variable which raises blood pressure, but does not modify the pressure natriuresis mechanism, *will not* cause chronic hypertension. Therefore, in order for sodium balance to be maintained in the face of a *persistent* increase in blood pressure, there must also be a shift in the pressure natriuresis mechanism such that it now operates at a higher arterial pressure. If this shift in pressure natriuresis did not occur, then increased arterial pressure would cause progressive loss of sodium and water until blood pressure was normalized. Given these facts, chronic hypertension is only possible if there is some renal dysfunction which shifts the pressure natriuresis mechanism such that sodium balance is established at a higher arterial pressure than normal. In fact, all forms of hypertension are characterized by some manifestation of renal dysfunction at some stage (Singh et al., 2010).

### Modulation of renal function by resveratrol

To our knowledge, there is currently no detailed information regarding the potential modulation of the pressure natriuresis mechanism by resveratrol in normal physiology or hypertension. Recently, Gordish and Beierwaltes (2014) demonstrated acute renal vasodilation in response to systemic infusion of resveratrol in anesthetized rats (Gordish and Beierwaltes, 2014). In this study, resveratrol appeared to cause a modest (8%) increase in renal blood flow and reduction in renal vascular resistance (18%) which was partially dependent on NO production and ROS scavenging (Gordish and Beierwaltes, 2014). While this is the only study specifically investigating renal actions of resveratrol, this work has limited clinical utility as it involves systemic infusion of resveratrol, is confounded by non-specific vehicle effects as well being performed in a normotensive animal model. Since hypertension is characterized by endothelial dysfunction and reduced renal perfusion (Tousoulis et al., 2012), agents such as resveratrol that increase local, intra-renal bioavailability of NO or reduce renal vasoconstriction may improve renal perfusion and thus ameliorate the resultant renal dysfunction that triggered, or contributes to the chronic hypertension. In light of the essential contribution of pressure natriuresis and renal function to the development and maintenance of chronic hypertension, investigation of the direct renal effects of resveratrol in hypertension is most pressing. Despite this lack of specialized information, there is however a wealth of experimental data with respect to the peripheral and cellular actions of resveratrol in hypertension which will now be discussed in the setting of oxidative stress as a putative mechanism in the development of this chronic condition. The potential impacts of these documented peripheral effects on renal function will also be reasoned when appropriate.

## Oxidative stress in the pathogenesis of hypertension

While the connection between free radicals and hypertension has been known since the 1960's (Romanowski et al., 1960), it was not until the 1990's that the role of oxidative stress in the development of hypertension began to be fully investigated (Nakazono et al., 1991). Evidence suggests that oxidative stress is a major cause of reduced bioavailability of endothelial NO in cardiovascular disease, including hypertension (Rajagopalan et al., 1996; Jia et al., 2010; Grande et al., 2011; Bhatia et al., 2012). NO signals the smooth muscle to relax, resulting in vasodilation, increased blood flow and a reduction in blood pressure. NO is synthesized endogenously from L-arginine, oxygen, and nicotinamide adenine dinucleotide phosphate (NADPH) by various nitric oxide synthase (NOS) enzymes. Increased production of ROS decreases NO bioavailability by direct inactivation, reduced oxidation of tetrahydrobiopterin (BH_4_; enzyme essential for NOS function) or inhibition of dimethylarginine dimethylaminohydrolase (DDAH; enzyme that degrades the NOS inhibitors, methylarginines). Antioxidants can reduce oxidative stress by direct scavenging of ROS and can directly improve endothelial function by reducing NADPH-oxidase activity or increasing superoxide dismutase (SOD) activity.

Early experiments demonstrated a reduction in blood pressure of Spontaneously Hypertensive Rats (SHRs) in response to administration of SOD (Nakazono et al., 1991), an important component of the endogenous antioxidant defense system. Increased production of ROS was also noted early on in rat Ang II-infused hypertension and this was attributed to activation of NADPH-oxidase a membrane-bound complex that can generate ROS (Rajagopalan et al., 1996). More recent studies show that ROS are important in blood pressure regulation which has led to rising interest in oxidative stress as a mediator in the pathophysiology of hypertension. In fact, most experimental models of hypertension exhibit indications of oxidative stress (Jia et al., 2010; Grande et al., 2011; Bhatia et al., 2012) and mice with a genetically engineered deficiency in ROS-generating enzymes have lower blood pressure than wild type mice (Haque and Majid, 2011). Ang II infusion in these mice also fails to produce hypertension (Haque and Majid, 2011). As a result, ROS are proposed to be causative in the development of hypertension due to the largely anti-hypertensive effect of inhibition of ROS-producing enzymes or the administration of antioxidants and ROS-scavengers (Lob et al., 2011). Conversely, pro-oxidants typically increase blood pressure. Although the relationship between ROS and blood pressure is clear in animal models, the linkage is less impressive in humans (Franco et al., 2007; Cottone et al., 2009) with no direct evidence of causality at present. However, despite this lack of data, it is known that diets rich in antioxidants or supplementation with antioxidants is largely beneficial for prevention and management of hypertension (Montezano and Touyz, 2012), although clearly further investigation in humans must be completed.

### Oxidative stress in cardiovascular and renal circulation

Potential sources of ROS that are involved in the pathogenesis of hypertension include: uncoupled NOS which generates superoxide rather than protective NO (Nishino et al., 2008); activation of NADPH-oxidase (Adlam et al., 2007); xanthine oxidoreductase and mitochondrial respiratory enzymes (Sedeek et al., 2009). The main endogenous antioxidants in the body are SOD, catalase, glutathione peroxidase, thioredoxin and peroxiredoxin, bilirubin and uric acid (Chen et al., 2001; Gongora et al., 2006; Goyal and Basak, 2010). Major non-enzymatic antioxidant systems include vitamins C, E, and glutathione, although at high levels, vitamins C and E can have pro-oxidant effects (Traber and Stevens, 2011). Bilirubin inhibits lipid oxidation and formation of oxygen radicals, acting as a physiological antioxidant and provides protection against cardiovascular disease (Lin et al., 2010). In fact, low levels of bilirubin are linked to hypertension, diabetes and metabolic syndrome, making it a potential biomarker of cardiovascular disease (Lin et al., 2010). Generally, low levels of endogenous antioxidants promote oxidative tissue damage to cardiovascular and renal systems which is implicated in the development of hypertension. For example, hypertensive humans exhibit lower plasma and cellular activities of SOD, catalase and glutathione peroxidase vs. normotensive controls (Redon et al., 2003). Because of the protective effect of antioxidants, there is intense interest in the application of natural or synthetic antioxidant molecules in therapeutic strategies geared toward the prevention and/or treatment of hypertension (Kizhakekuttu and Widlansky, 2010). In order to effectively target such treatments, an understanding of the mechanisms underlying oxidative-stress in hypertension is essential.

### Molecular mechanisms of oxidative stress in hypertension

#### Systemic

The cellular mechanisms which link oxidative stress and hypertension involve activation of redox-sensitive signaling pathways—signaling cascades that sense changes in cell oxidation/reduction (i.e., redox) status. This is noted especially in the vasculature where the bioavailability of the vasodilator NO is reduced and ROS production is increased. ROS such as superoxide and hydrogen peroxide act as second messengers and can activate mitogen-activated protein kinases (MAP kinases) tyrosine kinases, Rho kinase and transcription factors and can inactivate protein tyrosine phosphatases (Droge, 2002; Al Ghouleh et al., 2011; Zinkevich and Gutterman, 2011). ROS can increase intracellular Ca^2+^ concentration, favoring constriction of vascular smooth muscle for example. Up-regulation of pro-inflammatory gene expression and activity by ROS in addition to these modifications of cell signaling ultimately leads to endothelial dysfunction, reduced vasodilation and increased vasoconstriction (Lee and Griendling, 2008; Touyz and Briones, 2011), factors which can eventually increase TPR and blood pressure.

#### Renal

In light of the essential role of the kidney in the development of hypertension, it is also known that intra-renal oxidative stress is also detrimental. Activation of intra-renal redox-sensitive signaling pathways is associated with glomerular damage, proteinuria, excessive sodium and water retention as well as nephron loss (Wilcox, 2005); data generally support a “rightward” shift of pressure natriuresis to a higher pressure operating point (Cowley, 2008). As discussed, all of these factors are important processes in the development of hypertension as they ultimately affect renal excretory capability and alter blood volume. Blood flow in the renal medulla is now known to contribute to the normal function of pressure natriuresis and is, in fact, reduced in many forms of experimental hypertension (Cowley, 1992; Cowley and Roman, 1996). More recently, the importance of local medullary production of NO, superoxide, and hydrogen peroxide is recognized in pressure natriuresis and diuresis as well as disruption of this mechanism in renal injury and hypertension (Cowley, 2008).

In normal physiology, change in renal medullary blood flow is a key signal for the pressure natriuresis response and provides a crucial link to long term control of sodium excretion and blood pressure. Reduced medullary blood flow is observed in genetic forms of hypertension such as the SHR, where even very young (3–5 week) rats exhibited reduced medullary blood flow compared to controls; this blood flow was progressively reduced with age (Roman and Kaldunski, 1988). Since pressure-natriuresis was reduced at the very young age in the SHR, before the development of increased blood pressure, the reduction in medullary blood flow is suggested to contribute to a reduction of sodium excretory function and thus to the development of hypertension in SHR. Similarly, renal medullary blood flow was significantly reduced in Dahl salt sensitive rats within 24 h of increasing salt intake from 0.4 to 4.0% (Miyata and Cowley, 1999). If this reduction in medullary blood flow is prevented by intra-renal infusion of L-arginine (precursor of NO formation), the salt-induced hypertension was prevented (Miyata and Cowley, 1999). Although many factors affect medullary blood flow, NO is the most studied and it is clear that NO production in the renal medulla participates in regulation of blood flow and protects this critical region from ischemia-related injury (Cowley et al., 2003). It thus becomes apparent that mechanisms which compromise the normal bioavailability of NO within the kidney can reduce medullary blood flow and alter pressure natriuresis such that the development of hypertension is facilitated. Indeed, chronic administration of the NOS inhibitor L-NAME results in a 30% reduction in renal medullary blood flow with a reduction in sodium excretion and the subsequent development of chronic hypertension (Nakanishi et al., 1995). In terms of tubular mechanisms, inhibition of intra-renal NOS activity causes an immediate reduction of sodium excretion by reducing tubular sodium transport in the medullary thick ascending limb (Ortiz, 2000; Ortiz and Garvin, 2002). Interestingly, superoxide plays an important role in regulation of renal medullary blood flow and sodium excretion and is abundantly found in the renal medulla in normal conditions (Zou et al., 2001). Superoxide scavenging increases renal medullary blood flow and sodium excretion (Zou et al., 2001), whereas chronically increased levels of superoxide produce hypertension (Makino et al., 2002). This is mediated by a superoxide-induced increase in sodium reabsorption in the medullary thick ascending limb (Juncos et al., 2006). Sources of superoxide in the kidney are primarily from NADPH-oxidase and mitochondrial respiratory chain enzymes (Zou et al., 2001). In addition to increased superoxide in salt-sensitive hypertensive models, hydrogen peroxide levels are also increased with oxidative stress further augmented by NOS uncoupling (Taylor et al., 2006a,b).

In addition to these renal medullary processes, there is a generalized pathological remodeling of renal afferent and efferent arterioles in hypertension that compromises autoregulation and thus allows high blood pressures to damage the delicate glomerular structures (Palm and Nordquist, 2011). The renal vascular endothelium becomes dysfunctional as occurs in the extra-renal vasculature, however in the kidney, this results in a shift of the pressure natriuresis mechanism to higher blood pressures. Renal vasoconstriction begins to dominate resulting in hypoxia and subsequent fibrogenesis with tubular collapse, progressively reducing excretory capacity of the kidney (Palm and Nordquist, 2011). Oxidative stress is thought to play an integral role in this progression of kidney disease. Indeed, hypertensive rats have lower SOD and catalase activities and impaired renal vasodilation is improved by SOD treatment (Rajagopalan et al., 1996; Taylor et al., 2006a). SOD mimetic (Tempol) administration normalizes blood pressure, renal vascular resistance and reduces oxidative stress in SHRs (Schnackenberg et al., 1998; Schnackenberg and Wilcox, 1999, 2001). Elevated renal perfusion pressure itself can increase vascular ROS production via activation of NADPH-oxidase (Ungvari et al., 2003), while Ang II has been shown to also trigger intrarenal ROS production by inducing mitochondrial dysfunction with subsequent NADPH-oxidase activation and formation of peroxynitrite (Laursen et al., 1997; Doughan et al., 2008). This Ang II induced hypertension and mitochondrial dysfunction is attenuated by overexpression of the mitochondria-specific antioxidant enzyme thioredoxin 2 (Widder et al., 2009). These mitochondrial-targeted antioxidants inhibit superoxide production in the kidney, attenuate NADPH-oxidase activity and restore NO bioavailability (Dikalova et al., 2010). This prevents hypertension and improves endothelium-dependent vasodilation at the onset of Ang II infusion, highlighting the critical contribution of renal mitochondrial superoxide in the development of hypertension. In addition to these mechanisms, oxidative stress also induces inflammation and fibrosis within the kidney which ultimately facilitates the development of hypertension. In the presence of the SOD mimetic Tempol, rats exposed to a high-salt diet showed an amelioration of hypertension which is most likely linked to the prevention of renal inflammation and fibrosis by reducing renal oxidative stress and improving sodium excretion (Roson et al., 2011).

Renal NADPH-oxidase is also likely important for increasing oxidative stress in the vasculature and kidneys and makes a major contribution to the development of hypertension. Several NADPH-oxidase isoforms are present in the kidney (Nox 1, 2, 4, and 5) (Lassegue et al., 2012) and are major sources of intra-renal ROS (Chen et al., 2012), which are potent renal vasoconstrictors. This increased renal Nox activity and associated increase in ROS is known to participate in many cardiovascular and renal disorders, including hypertension (Chen et al., 2012). For example, Nox 1, 2, and 4 are increased in kidneys of SHRs or Ang-II infused hypertensive rats (Wingler et al., 2001; Wind et al., 2010; Wilcox, 2012; Lo et al., 2013). This pattern is not limited to rodent models since Nox 5 expression is also increased in renal proximal tubule cells from hypertensive humans compared to normotensive counterparts and may account for increased oxidative stress in these cells (Yu et al., 2011). It is also well known that intra-renal ROS production is increased in response to stimulation by the RAAS (Cuevas et al., 2013). Over-activity of the RAAS is implicated in hypertension and it has been shown that Ang II, via AT_1_R, activates NADPH-oxidase with the subsequent formation of superoxide (Chabrashvili et al., 2003). All components of NADPH-oxidase are expressed in kidneys of SHRs and p47^phox^ (subunit of NADPH-oxidase) is increased in all structures of the kidney including vasculature, macula densa and distal nephron compared to controls (Chabrashvili et al., 2002). This increase in p47^phox^ precedes the development of hypertension (Chabrashvili et al., 2002), further implicating renal NADPH-oxidase in this pathogenesis. Increased salt intake, which predisposes hypertension, has been shown to increase renal superoxide generation as well as NADPH-oxidase expression and reduces intrarenal SOD expression (Kitiyakara et al., 2003). Renal NOS uncoupling is also implicated in hypertension (Landmesser et al., 2003); the increase in superoxide production in hypertensive DSS rats on high salt diet is attributed to NOS uncoupling as is increased ROS production in hypertensive mouse aorta (Taylor et al., 2006b). Although NO bioavailability is definitely compromised in many regions of the body in hypertension, intra-renal oxidative stress and disruption of the NO system in the renal circulation alone has widespread impact on the entire cardiovascular system given its central role in regulation of excretion and also direct neurohormonal communication with other systems such as the brain and vasculature. The central role of the kidney in the pathogenesis of hypertension as well as the documented increase in kidney-specific oxidative stress make this an attractive target for resveratrol treatment, particularly in elucidating the potential mechanisms underlying the reported anti-hypertensive effects of this molecule.

#### Brain and sympathetic nervous system

Despite the overwhelming support for a central role of the kidney in the long-term regulation of blood pressure and development of hypertension, there exist criticisms of the original model proposed by Guyton and Coleman. The main crux of the argument against a central role for the kidney in hypertension resides in the belief that the central nervous system (CNS) modulates blood pressure via regulation of sympathetic outflow (Kishi et al., 2003, 2004; Chan et al., 2005; Osborn et al., 2009; Braga, 2010). While sympathetic control of vascular tone is indeed critical for short-term regulation of blood pressure, in the presence of a normally functioning kidney, any increase in blood pressure due to sustained neurally mediated vasoconstriction would be normalized by a concomitant increase in sodium and water excretion. In terms of the development of hypertension, it is not a question of whether the brain and sympathetic tone is involved in long-term blood pressure regulation, but rather *how* it is involved in this pathogenesis. In the arena of oxidative stress, brain-produced ROS have been documented and largely attributed to NADPH-oxidase in the hypothalamus and circumventricular organs (Fujita and Fujita, 2013). These regions are intimately involved in the control of sympathetic outflow and have thus, along with the rostral ventromedial medulla (RLVM) been implicated in the central control of the development of hypertension. Indeed, brain oxidative stress-mediated sympathoexcitation has been suggested in some hypertensive animal models and limited data in humans (Fujita et al., 1980, 2007, 2012; Kishi et al., 2004; Ye et al., 2006; Koga et al., 2008; Nagae et al., 2009; Oliveira-Sales et al., 2010; Purushothaman et al., 2011). Most importantly, it is key to remember that sympathetic outflow, while discrete, also can include activation of renal sympathetic nerve activity (RSNA) and ultimately modulate renal function. In addition, as mentioned earlier, via renal afferent nerve activity as well as release of hormonal mediators that are known to act at the circumventricular organs (i.e., Ang II) with sympathoexcitatory effects, the kidney is also able to relay information to the CNS, resulting in changes in sympathetic outflow and peripheral regulation of vascular tone (Converse et al., 1992; Campese and Kogosov, 1995; DiBona and Kopp, 1997). This is the most likely explanation for exciting results in human resistant hypertensive patients in which surgical ablation of renal nerves significantly reduced blood pressure (Krum et al., 2009; Bisognano et al., 2011; Schlaich et al., 2012) and also in patients in which bilateral carotid artery stimulation (which ultimately reduces renal sympathetic tone via the CNS) also significantly reduced blood pressure (Bisognano et al., 2011). Even though the brain and nervous system are involved in these treatment strategies, the final common pathway is modification of renal function with a subsequent reduction in blood pressure.

## Amelioration of oxidative stress and hypertension by resveratrol

### Molecular targets of resveratrol

The general anti-oxidant and superoxide-scavenging properties of resveratrol have been suggested as the basis of part of its protective effects on the cardiovascular system (Vidavalur et al., 2006). In addition, resveratrol has also been implicated in modulation of enzyme function and expression. These effects involve interaction of resveratrol with surface receptors and modulation of cell signaling, metabolic pathways, nuclear receptor activation and regulation of gene transcription and translation (Pervaiz and Holme, 2009). *In vivo*, resveratrol is a potent antioxidant and has been shown to upregulate NO synthesis, modulate endogenous antioxidants such as thioredoxin as well as heme-oxygenase pathways (Das et al., 2008; Dudley et al., 2009), heme-oxygenase being a precursor of bilirubin and thought to be protective against hypertension. In contrast, resveratrol is a relatively weak antioxidant *in vitro*, which suggests that it primarily modulates endogenous anti-oxidant systems to produce more robust effects *in vivo* (Das et al., 2008).

Sirtuins are one of the molecular targets of resveratrol and are members of the Sir2 (silent information regulator 2) family, a group of class III deacetylases (Albani et al., 2010). Mammals have seven different sirtuins, SIRT1-SIRT7. Among them, SIRT1, SIRT3, and SIRT6 are induced by calorie restriction conditions and are considered anti-aging molecules. Generally, sirtuins silence gene transcription and are protective in the setting of oxidative stress, being linked with increased expression of antioxidant enzymes and attenuation of age-dependent increases in heart weight, apoptosis, fibrosis and cardiac dysfunction (Alcendor et al., 2007). SIRT 1 is the most extensively studied and is thought to mediate the effects of resveratrol (Stiaccini et al., 2012). In fact, SIRT 1 deficiency, particularly in the setting of oxidative stress, has been implicated in diabetes, renal dysfunction and cardiovascular diseases such as hypertension (Kitada and Koya, 2013; Kitada et al., 2013). In line with this, caloric restriction, which normally activates SIRT 1 (Guarente, 2013) has been shown to reduce blood pressure in humans (Holloszy and Fontana, 2007) and prevent the development of hypertension as well as preserve mesenteric vascular compliance and endothelium-mediated vasorelaxation in SHRs (Dolinsky et al., 2010). Resveratrol is also deemed a “calorie restriction mimetic” (Chung et al., 2012) and may be activate similar signaling pathways, including SIRT 1 to exert its antihypertensive effects. Resveratrol significantly increases SIRT 1 expression in human renal cells, even at low doses (Stiaccini et al., 2012), suggesting a reno-protective effect via SIRT 1 amelioration of oxidative stress. Resveratrol also mediates its protective effects via the thioredoxin system (Maulik and Das, 2008), which is part of the body's endogenous antioxidant defense mechanisms and is integral for maintaining intracellular redox status. Altered function of this system has been implicated in hypertension (Maulik and Das, 2008) and although resveratrol can activate thioredoxin-2 which confers benefit in heart disease (Dudley et al., 2009), it is unclear whether this also has beneficial impact in hypertension. Resveratrol also indirectly activates heme-oxygenase-1 (HO-1) via increased NO production, since NO can induce expression of HO-1 (Son et al., 2013). Inhibition of HO-1 prevents resveratrol-induced phosphorylation of p38 MAP kinase b, Akt as well as blocks p38 MAP kinase an activation—all downstream mediators of resveratrol-induced cell signaling (Son et al., 2013). In addition to these mediators, resveratrol also activates the 5′ adenosine monophosphate-activated protein kinase (AMPK)/Akt/eNOS signaling pathway, which is important in the anti-hypertensive and anti-diabetic effects of resveratrol in models of Type 2 diabetes (Penumathsa et al., 2008). In addition, resveratrol restores mesenteric and cardiac eNOS activity and overall oxidative stress in this model (Miatello et al., 2005).

### Vascular remodeling as a target of resveratrol in hypertension

The vascular endothelium senses mechanical and hormonal stimuli and in response to an increase in these parameters, releases agents that modulate vasomotor function. Generally endothelial cells have a regulatory and protective role by generating vasorelaxing substances, namely NO. However, in the pathophysiological setting, vasoconstrictors such as ET-1 are released, contributing to an overall increase in total peripheral resistance with time. In the short term, vascular smooth muscle cells respond to mediators released by the endothelium and regulate blood vessel diameter. However, in the long term, these cells adapt via vascular remodeling. ROS mediate these adaptive and eventually pathophysiological processes, eventually contributing to the development of hypertension. Peripheral vascular effects of resveratrol may be more effective targets for preventative therapy, rather than treatment of established hypertension in which detrimental and often irreversible vascular remodeling has already occurred in multiple vascular beds. Activation of SIRT 1 may initiate the vascular actions of resveratrol since it is a key regulatory molecule in the control of vascular endothelial homeostasis, controlling angiogenesis, vascular tone and endothelial dysfunction (Mattagajasingh et al., 2007; Potente and Dimmeler, 2008). Overexpression of SIRT 1 paired with subsequent treatment with resveratrol decreased expression of Ang II AT_1_ receptors in rat vascular smooth muscle cells (Miyazaki et al., 2008). Similarly, AT_1_ receptor expression in mouse aorta is suppressed by resveratrol and Ang-II-induced hypertension is attenuated in these animals (Miyazaki et al., 2008). Although these studies support a mechanism by which resveratrol can limit vasoconstriction and ameliorate hypertension, they must be interpreted with caution since the aorta is a conduit vessel which does not contribute to vascular resistance and any sustained reduction in blood pressure likely also involves some renal effect as emphasized earlier, such as amelioration of renal vascular AT_1_ receptor expression and renovascular vasoconstriction. However, this concept is merely speculation and studies investigating these putative renal effects of resveratrol must be completed. Preservation of endothelial cell function has also been documented with physiological doses of resveratrol and appears to involve increased expression of eNOS and reduced expression of endothelin-1 (ET-1) as observed in human umbilical vein endothelial cells (Nicholson et al., 2008). Additional mechanisms include up-regulated expression of glutathione peroxidase, catalase and HO-1 observed in cultured rat arteries (Ungvari et al., 2007), processes which ultimate prevent oxidative stress and subsequent endothelial cell death and thus may signify an important target for resveratrol in early prevention and improvement of long-term cardiovascular function (Xia et al., 2008).

Rarefaction or gradual loss of functional capillaries has been implicated in the rise of total peripheral resistance in hypertension. Thus, formation of new capillaries via neovascularization or angiogenesis increases tissue blood flow (Maulik, 2006) reduces total peripheral resistance and is a potential target for anti-hypertensive therapy. Resveratrol has been shown to upregulate vascular endothelial growth factor (VEGF) and VEGF receptor expression in the ischemic rat myocardium in addition to upregulation of thioredoxin and HO-1 which, in combination, not only reduces oxidative stress, but promotes myocardial angiogenesis (Koga et al., 2008). This action of resveratrol, in conjunction with putative reno-protective actions represent a multi-target treatment approach with one drug that can potentially lower blood pressure by multiple mechanisms as well as reduce the need for surgical interventions such as angioplasty and bypass surgery with respect to improving cardiac function, which is often a concern in patients with established and chronic hypertension. In line with this, considering the increased risk of stroke in hypertension, the observation that resveratrol induces angiogenesis following cerebral ischemia (Dong et al., 2008) is also of benefit to repair brain damage after stroke and improve patient survival and quality of life.

### Direct peripheral vasorelaxation by resveratrol

Resveratrol is also known to cause direct vasorelaxation by actions on smooth muscle and endothelial cells. In a model of obesity hypertension, resveratrol prevented the expected increase in blood pressure and preserved endothelium-dependent relaxation of aortic rings (Aubin et al., 2008). Once again, this type of study must be interpreted with caution, not only because a conduit vessel was assessed, but also because administration of resveratrol before the onset of hypertension is more supportive of a preventative, rather than therapeutic role for this agent. Thus, experimental design and treatment timeline is a critical factor in assessing the utility of resveratrol in hypertension and may help explain the controversial findings in human and animal studies with regard to blood pressure control.

At the cellular level, resveratrol opens large conductance Ca^2+^-operated K^+^ channels on rat aortic smooth muscle, which mediates an increase in NO release from endothelial cells, resulting in vasorelaxation (Calderone et al., 2007). A K^+^-independent vasorelaxation has also been noted in response to resveratrol in mesenteric arteries, suggesting multiple cellular modes of vascular relaxation by this molecule (Gojkovic-Bukarica et al., 2008). Resveratrol also reduced agonist-induced increases in intracellular Ca^2+^ in rat aortic vascular smooth muscle cells, thought to involve direct inhibition of Ca^2+^ channels or depletion of intracellular Ca^2+^ stores (Campos-Toimil et al., 2007). However, in endothelial cells, resveratrol increased agonist-induced intracellular Ca^2+^ release which triggers NO synthesis and decreases Ca^2+^ sensitivity, and thus contractility, of adjacent smooth muscle cells (Buluc and Demirel-Yilmaz, 2006). Additional mechanisms of direct vasorelaxation by resveratrol include increased NOS expression in vascular endothelial cells and activation of guanylate cyclase and increased cGMP-mediated relaxation (Klinge et al., 2005; El-Mowafy et al., 2008). In light of recent data supporting direct NO release from vascular smooth muscle cells, independent of the endothelium (Charpie and Webb, 1993; Cheah et al., 2002; Buchwalow et al., 2004, 2008), as well as evidence showing that circumferential stretch of the vasculature (as would occur in hypertension) promotes ROS formation (Haga et al., 2007; Thomas et al., 2008; Anwar et al., 2012), it is possible that resveratrol may additionally act directly on these cells to produce vasorelaxation. Indeed, data published this year indicate a role for resveratrol in the reduction of oxidative stress in vascular smooth muscle cells via suppression of ROS generation, Akt-phosphorylation, p38 MAPK phosphorylation and lKB-α and NF-KB activities (Guo et al., 2014); direct activation of AMPK and suppression of Ras homolog gene family member A (RhoA)/Rho associated Kinase (ROCK) in murine vascular smooth muscle cells also prevents Ang II hypertension (Cao et al., 2014).

Continued studies on the mechanisms of action of resveratrol at the molecular level are certainly important in order to determine its full therapeutic potential. However, with respect to hypertension, it must be emphasized that true resistance vessels, rather than conduit vessels must be studied in order to produce physiologically meaningful results. In addition, it is imperative that research efforts must include focus on renal vascular effects of resveratrol. Likely, mechanisms similar to those just described are at play in the renal circulation in order to produce the often-reported sustained reductions in arterial pressure. In fact, perhaps by studying the renal vascular actions of resveratrol, we may discover targets for treatment of established hypertension via the amelioration of renal dysfunction. As it stands, the peripheral vascular effects of resveratrol are most likely best applied for the early prevention of remodeling and subsequent hypertension, as these vasorelaxant effects of resveratrol alone most certainly will not reduce blood pressure in established hypertension without a parallel improvement in renal function.

### Efficacy of resveratrol administration in experimental hypertension

Grape-derived products including resveratrol have been shown to reduce blood pressure in several animal models of hypertension including SHR (Miatello et al., 2005; Peng et al., 2005; Dolinsky et al., 2013), L-NAME (Bernatova et al., 2002), DOCA-salt (Jimenez et al., 2007; Chan et al., 2011), partially nephrectomized (Liu et al., 2005; Bhatt et al., 2011), Goldblatt 2 kidney, 1 clip (Toklu et al., 2010) and Ang II administration (Sarr et al., 2006; Dolinsky et al., 2013). In addition to lowering blood pressure in rats (Mizutani et al., 1999; Diebolt et al., 2001; Bernatova et al., 2002), red-wine polyphenols also reduce end-organ damage which has been attributed to a reduction in overall oxidative stress and endothelial dysfunction (Perez-Vizcaino et al., 2009). Resveratrol administration reduces ET-1 and Ang-II concentrations and increases NO concentration which helps protect against increased blood pressure and cardiac hypertrophy (Liu et al., 2005). In a model of malignant hypertension (SHR + L-NAME treatment), resveratrol not only reduced blood pressure by 45 mmHg, it also reduced aortic blood flow and aortic vascular resistance (Milanovic et al., 2010). The mechanism for this change was attributed to a resveratrol-mediated increase in the antioxidant enzyme glutathione and the prevention of disintegration of endothelial cells and remodeling/necrosis of vascular smooth muscle (Milanovic et al., 2010). The main conclusion of this study was that resveratrol likely reduced arterial pressure by reducing total peripheral resistance (Milanovic et al., 2010). In our laboratory, we have shown that early treatment of both SHR and Ang II-infused hypertensive rodents prevents the expected rise in systolic pressure, improves vascular function and prevents cardiac hypertrophy (Dolinsky et al., 2013). Mesenteric arterioles, which are true resistance vessels and contribute to total peripheral resistance, exhibited increased eNOS phosphorylation with resveratrol treatment which was mediated by resveratrol-induced activation of the AMPK-eNOS signaling cascade (Dolinsky et al., 2013). However, while some investigators report significant reductions in blood pressure in rodent models of hypertension, others report no change in this parameter in similar models (Mizutani et al., 2001; Rush et al., 2007; Dolinsky et al., 2009; Thandapilly et al., 2010; Rimbaud et al., 2011). This variability in blood pressure response to resveratrol may be attributed to different mechanisms of hypertension in each model, or differences in experimental design, point of initial treatment (i.e., pre- or post-onset of hypertension) as well as dose, route, and length of resveratrol treatment. In this light, we have observed that initiation of resveratrol treatment after the establishment of hypertension in SHRs does not reduce blood pressure (unpublished observation). Focus on the peripheral vasodilator properties of resveratrol, particularly after establishment of hypertension, is also misleading and unlikely to produce a lasting reduction in blood pressure. Targeted renal administration of resveratrol in the experimental setting may more effectively and consistently prevent or perhaps even reverse hypertension, however this has yet to be tested.

Resveratrol also participates in the modulation of autonomic tone. Overactivity of autonomic tone has been implicated in hypertension; particularly as it pertains to RSNA and control of renal perfusion and excretion (Muirhead, 1993; Singh et al., 2010). Following 30 day treatment with L-NAME or L-NAME+resveratrol, rats exhibited reduced sympathetic vascular tone and increased baroreflex sensitivity (Dillenburg et al., 2013). Although this study did not show a parallel reduction in blood pressure, the increase in baroreflex sensitivity suggests that resveratrol may contribute to a reduction in RSNA since activation of the arterial baroreflex would be expected to occur at lower arterial pressures than in the absence of treatment. Although this improvement by resveratrol was not enough to prevent the hypertension, it does indicate that the autonomic nervous system and baroreflex may be potentially fruitful targets, particularly in combination with traditional anti-hypertensive medications such as angiotensin receptor blockers (ARBs) or angiotensin converting enzyme inhibitors (ACEi) which have more impressive blood pressure lowering effects. In this scenario resveratrol can potentially reduce RSNA and help preserve renal perfusion and perhaps slow the progression of hypertension by protection of renal function and restoration of baroreflex sensitivity. In agreement with these results, we have observed a reduction in basal sympathetic tone in conscious SHRs receiving resveratrol for 8 weeks in the absence of a reduction in blood pressure (unpublished observation). Similarly, central injection of resveratrol into the RVLM, dose dependently reduced heart rate and RSNA in anesthetized rats, which was dependent on NO (Ma et al., 2008) and gives further support for a role of RSNA suppression by resveratrol. While this study also reported a dose dependent reduction in blood pressure, this result must be interpreted with caution as the effect was observed in normotensive rats under anesthesia (Ma et al., 2008). These studies highlight the link between the central nervous system and the eventual development of hypertension via centrally-mediated increases in RSNA. In this scenario, it is possible that central administration of resveratrol is protective against brain oxidative stress-induced increases in central sympathetic outflow, which is supported by evidence in a rat model of stroke and other studies showing complete quenching of superoxide in the brain by resveratrol (Mokni et al., 2007), with subsequent prevention of the development of hypertension (Subramanian et al., 2011).

As emphasized, renal-specific effects of resveratrol in the setting of chronic hypertension has not yet been thoroughly investigated. However, there is limited evidence for reno-protective effects of resveratrol in other areas of research that may be beneficial in hypertension. Oral resveratrol has been shown to attenuate hyperglycemia-induced oxidative stress in diabetic rats and this was associated with normalization of renal function and increased renal anti-oxidant activity (Palsamy and Subramanian, 2011). Liang et al. (2014) recently demonstrated the inhibition of renal oxidative stress and renal interstitial fibrosis by resveratrol, which may have application in the preservation of renal structures in hypertension (Liang et al., 2014). Human hemodialysis patients also show improved anti-oxidant activity following consumption of a diet rich in polyphenols (Castilla et al., 2006, 2008). There is also growing interest in the use of resveratrol for treatment and prevention of both acute kidney injury and chronic kidney disease. In a model of sepsis-induced kidney injury, renal microvascular failure is associated with the excessive generation of ROS. Given the ROS-scavenging properties of resveratrol, this molecule has therapeutic potential in this condition. Indeed, resveratrol reversed the sepsis-induced progressive decline in renal capillary perfusion in a dose-dependent manner with a parallel increase in renal blood flow and glomerular filtration rate in the absence of any change in systemic pressure (Holthoff et al., 2012). ROS was reduced in the renal tubules which contributed to the increase in renal function and overall survival with resveratrol treatment (Holthoff et al., 2012). This study reveals a dual renal mechanism of resveratrol to both scavenge ROS and prevent further renal injury and also restore renal microcirculation and renal perfusion which ultimately preserves renal excretory capability and prevents excessive retention of sodium and water—factors which increase blood volume and lead to chronic hypertension as discussed.

Although systemic mechanisms involved in sepsis differ from those in hypertension, both conditions are characterized by areas of local renal microcirculatory hypo-perfusion and areas of tissue hypoxia which lead to a pro-oxidant microenvironment favoring generation of ROS and are viable targets for treatment with resveratrol (Walker et al., 2001; Lundy and Trzeciak, 2009; Hollenberg, 2010; Wang et al., 2011). *In vitro*, resveratrol is a potent scavenger of peroxynitrite and thus protects tubular epithelial cells from injury (Holthoff et al., 2010). Resveratrol treatment also reduced ischemia-reperfusion injury of the rat kidney via its antioxidant properties and also reduced glomerular collapse in this setting (Bertelli et al., 2002). While ROS production is increased in renal afferent arterioles in response to increases in intraluminal pressure (Ren et al., 2010), it is not known whether resveratrol can prevent or ameliorate this. However, resveratrol does induce vasorelaxation in isolated renal arteries from normal and diabetic rats *in vitro* in the only published report in this area (Gojkovic-Bukarica et al., 2012), and may potentially involve reduction of ROS, which are known renal vasoconstrictors (Just et al., 2008; Lai et al., 2011). In support of this possibility, in 5/6 nephrectomized rat model, resveratrol attenuated the increase in blood pressure and preserved normal renal function as observed by a reduction in proteinuria, prevention of glomerulosclerosis and increased renal NO bioavailability as shown by increased urinary content of NO metabolites. The NO dependent-nature of the mechanism was demonstrated by complete prevention of these effects with co-administration of L-NAME (Chander and Chopra, 2006).

## Feasibility of resveratrol therapy in human hypertension

Although there is overwhelming experimental support for oxidative stress in the pathogenesis of hypertension, there is no solid evidence for a causative role of oxidative stress in human hypertension (Rodrigo et al., 2007). ROS are increased in patients with various forms of hypertension such as essential, renovascular, malignant, salt-sensitive, and pre-ecclampsia (Raijmakers et al., 2004; Ward et al., 2004; Rodrigo et al., 2007); hypertensive patients also exhibit increased plasma hydrogen peroxide that has even been detected in normotensive individuals with a family history of hypertension (Lacy et al., 1998, 2000). In this latter patient population, a genetic component may predispose for increased endogenous ROS production and as such, early antioxidant treatment in identified individuals may be of valuable preventative benefit. Hypertensive humans also exhibit reduced endogenous anti-oxidant defense mechanisms noted as reduced activity and tissue content of vitamins A, C, and E, SOD, glutathione, peroxidase and catalase; increased NADPH-oxidase activity and ROS in resistance arteries is also documented in these patients (Lacy et al., 2000; Touyz and Schiffrin, 2001; Caner et al., 2006; Simic et al., 2006; San Jose et al., 2008; Wang et al., 2009). Notwithstanding the comments above, there is some support for the potential use of antioxidants in the prevention and treatment of human hypertension. However, the number of clinical trials are quite limited and certainly more work must be completed in this area. Of the existing clinical trials investigating antioxidant treatment, findings are inconsistent and these studies are largely inconclusive.

Related to clinical trials involving the use of antioxidants, acute resveratrol administration in humans induces a transient increase in circulating NO, matched with a reduction in blood pressure and an increase in heart rate as well as an increase in endothelium-dependent vasodilation as measured by increased brachial artery blood flow (Hashimoto et al., 2001; Matsuo et al., 2001; Wong et al., 2011). Other studies report no change in blood pressure after resveratrol treatment in man (Pollack and Crandall, 2013; Poulsen et al., 2013). There is only one report of the acute effect of red wine on regional blood flow and sympathetic tone in man (Spaak et al., 2008). However, the dose provided in that study was extremely low (1–2 glasses of red wine) and no changes were noted between red wine and ethanol alone, excluding any independent physiological effects of polyphenols (Spaak et al., 2008). Only recently have results from more clinical trials on resveratrol become available. Resveratrol has anti-inflammatory and anti-oxidant effects in smokers (Bo et al., 2013), Type 2 diabetics (Brasnyo et al., 2011) and normal individuals (Ghanim et al., 2010, 2011) and also mimics the beneficial effects of caloric restriction in healthy, obese men (Timmers et al., 2011). However not all clinical trials with resveratrol are as impressive. 1 year resveratrol supplementation in hypertensive men with Type 2 diabetes mellitus did not improve hypertension but did reduce markers of inflammation and expression of pro-inflammatory cytokines (Tome-Carneiro et al., 2013). Notably, none of these studies reported adverse effects of resveratrol administration. Semba et al. (2014) measured urinary resveratrol metabolites in aged men and women of the Chianti region in Tuscany and concluded that normal dietary levels of resveratrol are not correlated with all-cause mortality (Semba et al., 2014). This result is not surprising, as normal levels of resveratrol in the diet are almost negligible and this study does not negate the fact that standardized, therapeutic doses of resveratrol may have beneficial impact on human health. There is also a lack of resveratrol studies involving young, healthy subjects. Most trials are focused on older patients with underlying disease processes which also makes it difficult to demonstrate the role of resveratrol as a preventative therapeutic option. Following our discussion outlining the critical role of the kidney in the development of hypertension, there are also no studies that have yet been reported that investigate the effect of resveratrol on renal function in man. Generally, disappointing results are likely related to the type of antioxidant compound used as well as dose and regimen. Currently, antioxidant supplementation is not formally recommended for the prevention/management of hypertension, but most guidelines do encourage the general population to consume a diet centered on more anti-oxidant-rich fruits, vegetables, and whole grains, as well as participation in regular exercise which lowers oxidative stress (Lopes et al., 2003; Adams et al., 2005; Agarwal et al., 2011; Rabi et al., 2011). There is most certainly room for resveratrol supplementation in these recommendations considering the demonstration of resveratrol as an exercise mimetic. In addition, classic anti-hypertensive treatments such as β-blockers, ARBs ACEi and Ca^2+^ channel blockers may have antioxidant effects that operate through direct antioxidant actions or stimulation of NO (Chen et al., 2008; Whaley-Connell et al., 2009; Weseler and Bast, 2010), further boosting the efficacy of combination treatment with resveratrol.

## Conclusion

**Figure 1:**
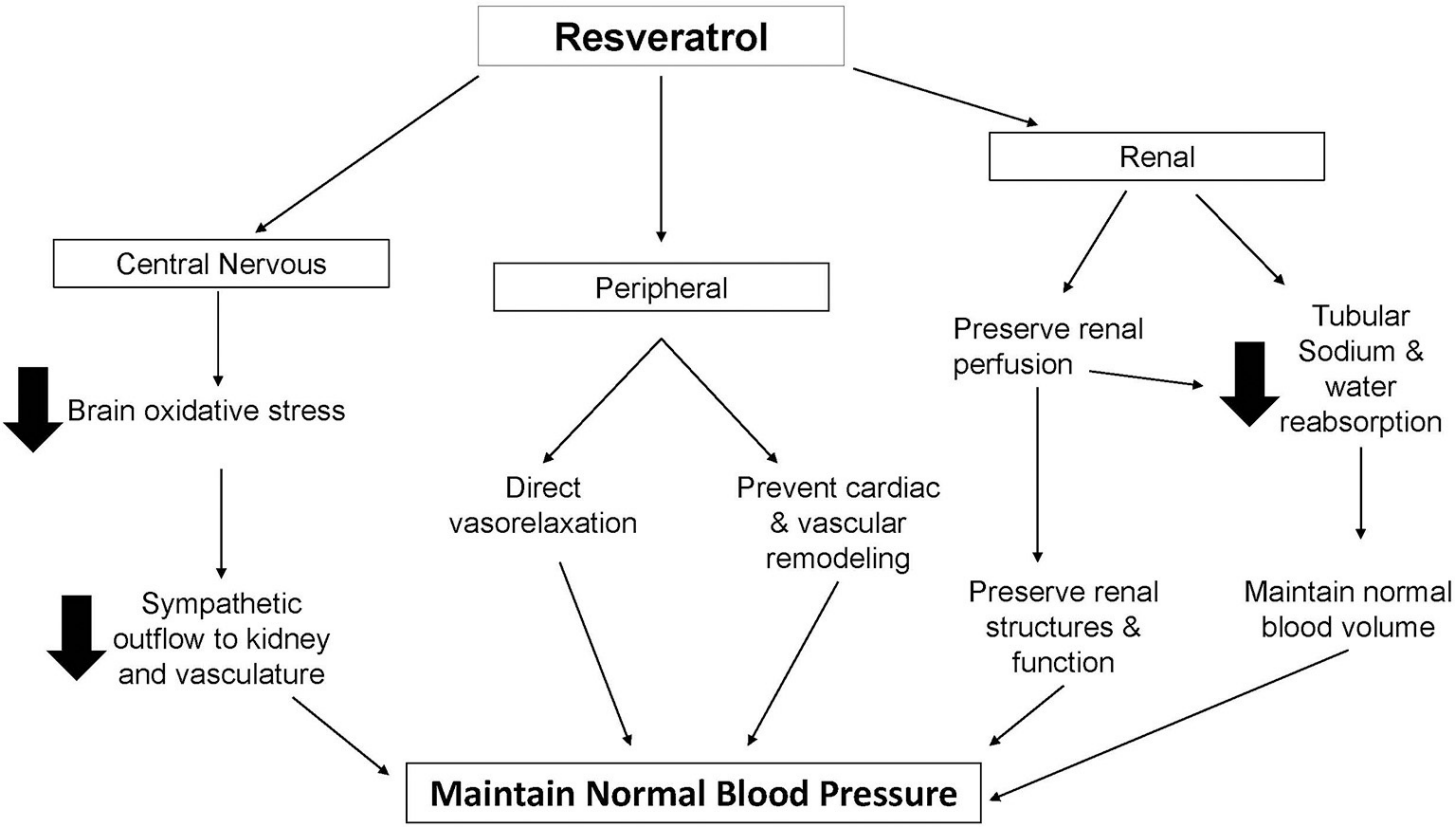
**Schematic representation of proposed antihypertensive actions of resveratrol**.

ROS play an important role in physiological regulation of cardiovascular and renal processes via redox-sensitive signaling pathways. However, when the systemic and renal generation of ROS exceeds the body's natural anti-oxidant defense capacity, ROS can cause significant tissue damage as well as modulate central, peripheral and renal mechanisms that result in cardiovascular and renal adaptations which ultimately initiate the development of hypertension. Amelioration of oxidative stress has thus been of interest in the prevention and treatment of hypertension and the use of natural anti-oxidant compounds has been intensively studied in recent years. Despite this concentrated interest in the application of molecules such as resveratrol in the treatment of hypertension, there has been virtually no investigation of the specific renal effects of such treatments. Almost all scientific efforts have been directed toward the peripheral and largely vasodilator properties of resveratrol. From a physiological standpoint this is concerning, given the established central role of the kidney in the long-term regulation of arterial pressure and development of chronic hypertension. In light of the “infinite gain” of the kidney to maintain normal blood volume and pressure, it is difficult to reconcile reported reductions of blood pressure and/or prevention of hypertension with exclusively extra-renal processes related only to reduction of cardiac output and total peripheral resistance. Many studies also draw conclusions based on investigation of conduit vessels such as the aorta which do not contribute meaningfully to total peripheral resistance and thus such results must be interpreted with caution. If resveratrol does not somehow influence the many facets of renal function, then any reduction in blood pressure via peripheral mechanisms observed with this treatment would be merely transient. As such, we propose that in addition to documented central and peripheral vascular actions of resveratrol, modulation of renal structure and function by this molecule may also be involved to produce anti-hypertensive protection and benefit (Figure 1). Exclusive focus on non-renal actions of resveratrol without consideration of the fundamental contribution of renal dysfunction to the initiation, development and maintenance chronic hypertension may be a factor accounting for the controversial and sometimes disappointing experimental and clinical results. The actions of resveratrol in the kidney, particularly in the setting of hypertension, is merely speculation at this time and this represents a much needed and potentially fertile area of future research.

### Conflict of interest statement

The authors declare that the research was conducted in the absence of any commercial or financial relationships that could be construed as a potential conflict of interest.
